# How Old Is a Man at Forty?

**Published:** 1921-01-08

**Authors:** 


### HOW OLD IS A MAN AT FORTY?

Not long ago we saw published an article by a physician advocating that every man should be thoroughly examined at forty, especially as to the condition of his arteries, heart, kidneys, and liver, with a view to finding out his real age as against his nominal age as reckoned in years from the date of his birth. It is pointed out that "everyone has two ages, the one recorded in his certificate of birth, and the other, far more vital and accurate, the age of his tissues." The examination is to be of a special nature and carried out by a physician who is in a position to utilise the latest appliances of medical science, and when the age of the tissues is determined there would be great benefits accruing, since "he would know what it would be advisable for him to do in such essential matters as climate, residence, diet, drink, clothing, work, rest, play, exercise, sleep, and recreation."

### A First Glance.

On the first glance this proposal seems a most useful one. But a little consideration will we think convince most people who know the British race that they will never submit themselves as long as they feel well, in any numbers worth mentioning, to such an examination, and we have, further, very grave doubts as to the possibility from a scientific point of view of finding out anything but the somewhat advanced cases of degeneration, and, when found out, of utilising the information for the benefit of the individual in any but a very small percentage of the cases examined.

Leaving the question of heredity or stock out of account, it may be said that the foundations of the health of the individual are laid in the first sixteen or seventeen years of life, *i.e.*, during the school period and up to the time when growth ordinarily ceases. It is during this period that most infections are acquired, habits are formed, and information stored which profoundly affect the after life of the individual. It is during this period that defects revealed by examination can be remedied, and it is during this period that the instruction given in bodily health and hygiene prevents the recipient from adopting a mode of life which may lead to premature degeneration of his tissues. It must be admitted that some occupations are much healthier than others, but making full allowance for this it will generally be found that, taking the population in the bulk, it is the habits of the individual rather than his work which determine the state of his arteries and heart at forty. If after twenty a man over-indulges in food or alcohol and acquires syphilis, that great cause of premature arterial degeneration, do not blame his work, rather blame his defective education before twenty!

Assuming next that the examination has taken place at forty, what is going to happen? If the subject is perfectly healthy he is told to "carry on." If he shows premature degeneration of any arteries and is "well off," he may alter his mode of life according to the medical findings, much to the patient's benefit. But suppose, on the other hand, he belongs to the working classes, has been trained to and spent his life at a certain occupation and is still capable of doing this, though not so vigorously as at, say, twenty-five, how is he to change his occupation? Anyone acquainted with the majority of working men knows how difficult it is to change an occupation even for such a disabling disease as tuberculosis, and how much more difficult will it be for such an indefinite complaint, as, say, a little arterio-sclerosis?

Further, one has to take into account the psychical effect of an announcement of premature decay with the possibility of early death on a man who, but for this special examination, felt in passable health and under no necessity of consulting a physician in the ordinary way.

### Another and Better Method.

Now, while we feel it our duty to point out the insuperable difficulties of this plan, we would like at the same time to point out another method by which the same end would be attained without unnecessary work and dislocation of the community. It is a wise provision of nature, and one which may be taken as axiomatic, that structural degenerations of any of the vital tissues very soon give rise to certain early symptoms of disease. Or we may put it another way, that offences against the laws of health very soon give rise to early symptoms of disease which, if not immediately attended to—as indeed they are most often not—will end in irreparable structural alteration. It matters not in practice which view of the origin of the "early symptom" we take. How important these early symptoms may be, Sir James MacKenzie has shown us in the case of heart disease, and no doubt the same holds good for other diseases, so if the public could be educated—and we look forward to the accomplishment of this—to consult their family physician, be he panel or private practitioner at the onset of these early symptoms, an immense amount of suffering and disablement could be saved, the onset of more serious symptoms avoided, and instead of prescribing some new occupation the patient could be given such advice and comfort as would enable him to add many years to his working life in the occupation he understands. Of course, this needs a certain amount of re-education of the practitioner or rather a readjustment of his ideas. At present his patients, with few exceptions, keep away from him till disease is well developed, and he gets little chance of studying the earliest symptoms except from the statements of the patient about the mode and time of onset; and these are, in the majority of cases, notoriously unreliable.

The British public moves slowly, but we hold that the plan here outlined contains a more practical solution of the question, and one which fits in better with the general scheme of preventive medicine.

